# A New Mechanistic Insight Into Macro‐Reentrant Atrial Tachycardia in a Patient With Modified Fontan Circulation

**DOI:** 10.1002/joa3.70334

**Published:** 2026-04-08

**Authors:** Peter C. Murray, Intisar Ahmed, Krishnakumar Nair

**Affiliations:** ^1^ Toronto General Hospital University Health Network Toronto Ontario Canada

## Abstract

Fontan flutter: an unusual example of an atrial tachycardia circuit in a patient with atrio‐pulmonary connection (APC) conduit. The tachycardia circuit propagates around the base of the conduit (encircled by the arrows, showing direction of propagation from earliest [red] to latest [purple]). Radiofrequency ablation was delivered (red dots) and tachycardia terminated on ablation at the green arrow.

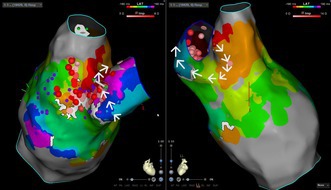

A 46‐year‐old man with complex congenital heart disease was brought for ablation of symptomatic atrial tachycardia due to recurrent triggering of decompensated heart failure. Anatomy posed a significant challenge in terms of access and mapping; d‐transposition of the great arteries, criss‐cross heart, and unrestrictive inlet VSD, corrected in infancy with a modified Fontan conduit for right atrial venous return to the main pulmonary artery (APC). Additionally, femoral veins were inaccessible due to previous DVT.

Electro‐anatomical mapping and ablation were performed, with the aid of intracardiac echo, through a short epicardial deflectable sheath via the right internal jugular vein, with a decapolar reference catheter in the esophagus.

This case demonstrates classical mechanisms of re‐entrant atrial tachycardia around an anatomical barrier, propagated by a zone of slow‐conducting scar. In order to create a line of block, it was necessary to deliver ablation lesions to the cavo‐atrial junction, in the vicinity of the sinus node; initial junctional rhythm recovered to sinus rhythm shortly after. Innovative access, support and reference electrogram enabled ablation of this novel substrate, termed the “cavo‐conduit isthmus” due to its mirror imaging of the classical cavo‐tricuspid isthmus (Figures 1, 2, 3, 4, 5).

**FIGURE 1:**
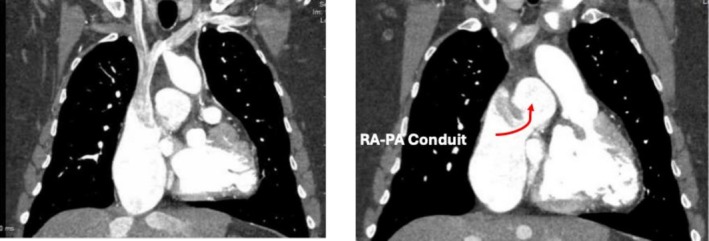
Contrast enhanced, ECG gated CT thorax in the coronal plane. The left panel shows SVC drainage into the RA conduit as well as contrast reflux into the intrahepatic IVC. The image on the right shows the conduit connection with the main pulmonary artery.

**FIGURE 2:**
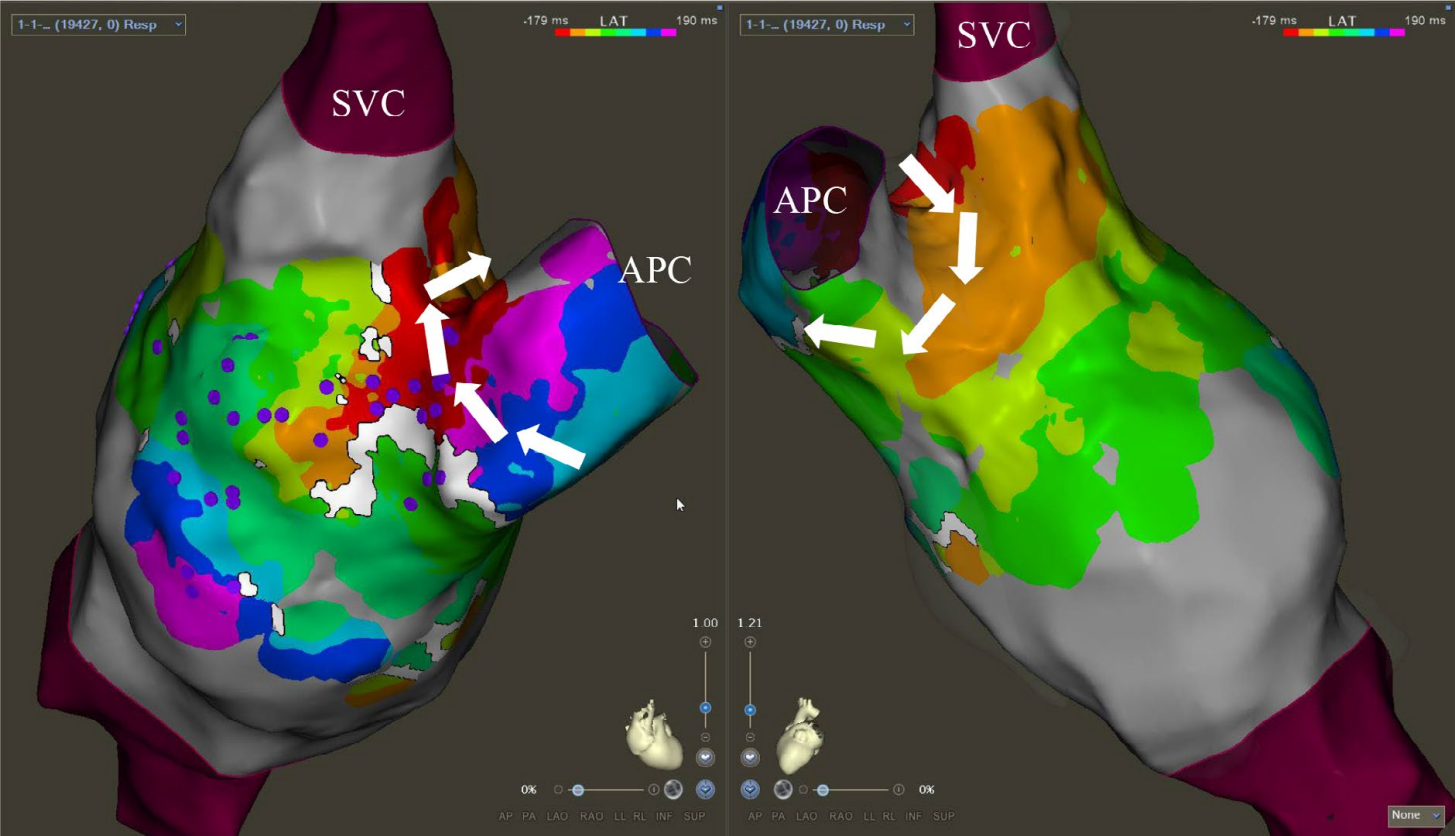
Isochronal activation map of right atrium viewed from anteriorly (Left) and posteriorly (Right) showing tachycardia circuit propagating around the conduit in a counter‐clockwise direction as viewed from caudal angle (white arrows) from earliest (red) posteriorly over the cavo‐conduit isthmus (orange), then around the septal aspect of the conduit in a posterior to anterior direction (yellow–green) to the anterior aspect (blue–purple), meeting the earliest activation again (red). APC, atrio‐pulmonary conduit; SVC: superior vena cava.

**FIGURE 3:**
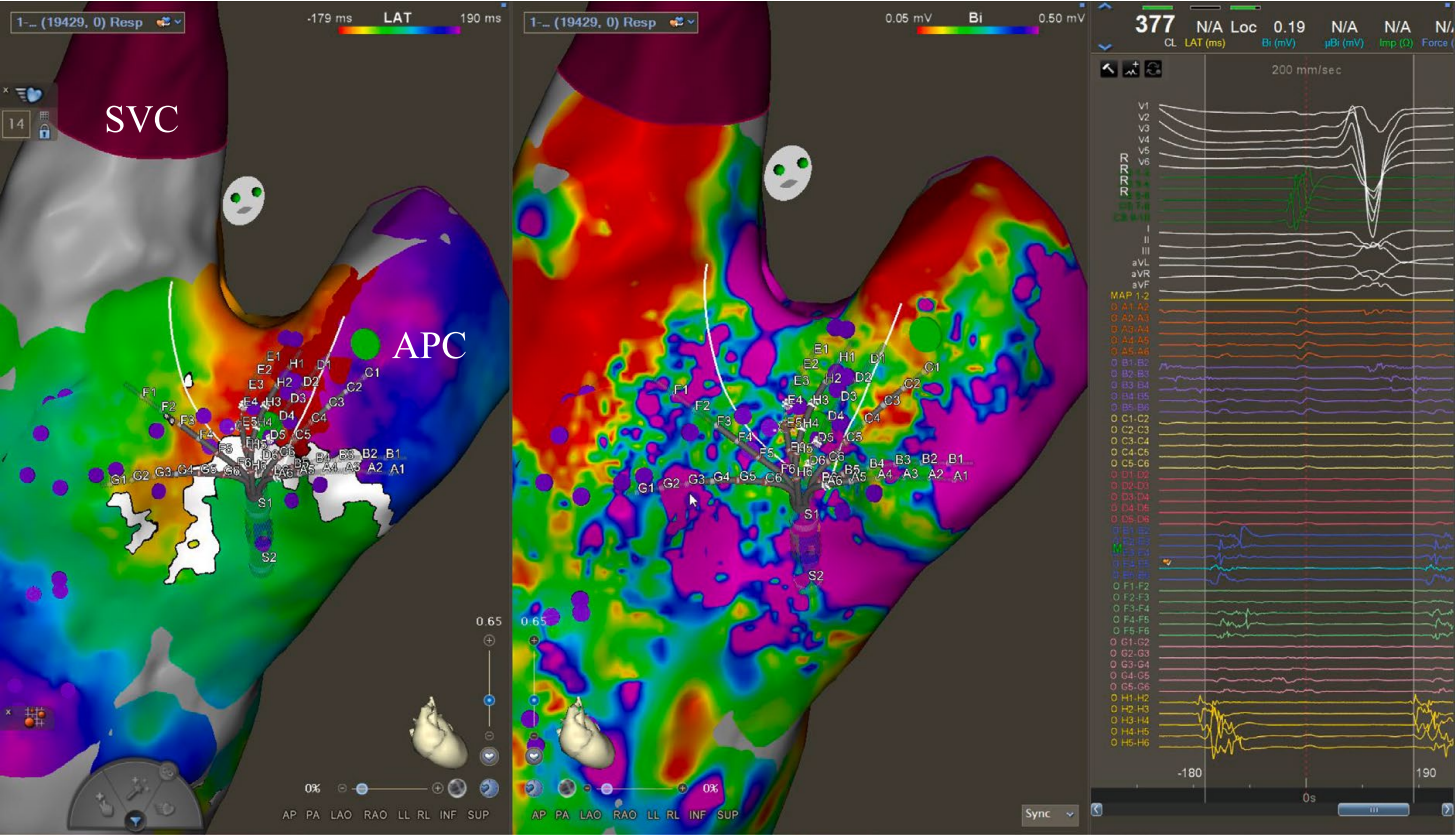
Anterior view of the “Cavo‐Conduit Isthmus”. Left: A zone of slow conduction is seen at the base. Centre: Low voltage scarring in this region. Right: Highly fractionated pre‐systolic signals on the mapping catheter in the region of the presumed isthmus.

**FIGURE 4:**
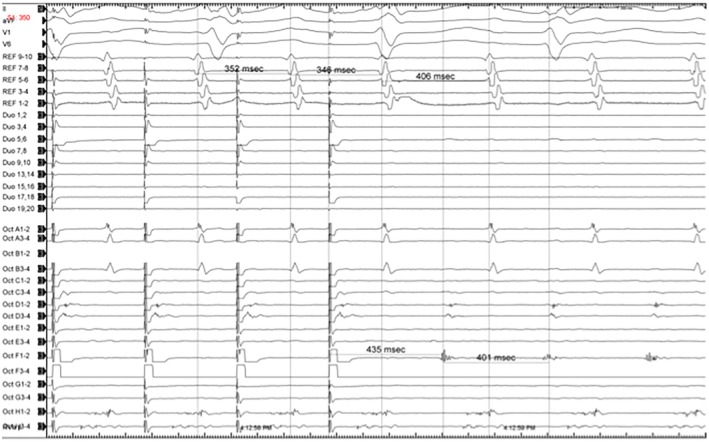
Entrainment from the presumed isthmus at 350 ms yields PPI‐TCL of 34 ms.

**FIGURE 5:**
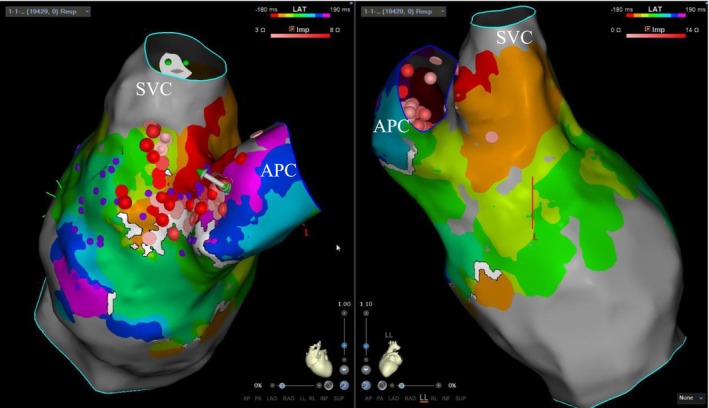
Ablation line (red dots) joining conduit to SVC, incorporating dense scar at the base (Figure 3). RF application to 40 W. TCL delays gradually as the line is drawn, terminating on the application shown (green pointer indicates tip of RF catheter).

## Conflicts of Interest

The authors declare no conflicts of interest.

## Data Availability

The data that support the findings of this study are available from the corresponding author upon reasonable request.

